# Thromboelastography parameters in diagnosing periprosthetic joint infection and predicting reimplantation timing

**DOI:** 10.1186/s12891-021-04578-x

**Published:** 2021-08-13

**Authors:** Tao Yuan, Yi Wang, Shui Sun

**Affiliations:** 1 Department of Joint Surgery, Shandong Provincial Hospital, Cheeloo College of Medicine, Shandong University, Jinan, 250012 Shandong China; 2 Department of Joint Surgery, Shandong Provincial Hospital Affiliated to Shandong First Medical University, Jinan, Shandong China

**Keywords:** Thromboelastography, Periprosthetic joint infection, Coagulation-related biomarkers, Two-stage revision

## Abstract

**Background:**

Coagulation-related biomarkers are drawing new attention in the diagnosis of periprosthetic joint infection (PJI). The thromboelastography (TEG) assay provides a comprehensive assessment of blood coagulation; therefore, it could be a promising test for PJI. This study aims to assess the value of TEG in diagnosing PJI and to determine the clinical significance of TEG in analysing reimplantation timing for second-stage revision.

**Methods:**

From October 2017 to September 2020, 62 patients who underwent revision arthroplasty were prospectively included. PJI was defined by the 2011 Musculoskeletal Infection Society criteria, in which 23 patients were diagnosed with PJI (Group A), and the remaining 39 patients were included as having aseptic loosening (Group B). In group A, 17 patients completed a two-stage revision in our centre. C-reactive protein (CRP), erythrocyte sedimentation rate (ESR), D-dimer, and TEG parameters (clotting time, α-angle, MA [maximum amplitude], amplitude at 30 min, and thrombodynamic potential index) were measured preoperatively in all included patients. In addition, receiver operating characteristic curves were used to evaluate the diagnostic value of these biomarkers.

**Results:**

ESR (area under curve [AUC], 0.953; sensitivity, 81.82; specificity, 94.87) performed best for PJI diagnosis, followed by MA (AUC, 0.895; sensitivity, 82.61; specificity, 97.44) and CRP (AUC, 0.893; sensitivity, 82.61; specificity, 94.74). When these biomarkers were combined in pairs, the diagnostic value improved compared with any individual biomarker. The overall success rate of the two-stage revision was 100%. Furthermore, ESR and MA were valuable in determining the time of reimplantation, and their values all decreased below the cut-off values before reimplantation.

**Conclusion:**

TEG could be a promising test in assisting PJI diagnosis, and a useful tool in judging the proper timing of reimplantation.

## Background

Periprosthetic joint infection (PJI), one of the most troublesome complications of total hip or knee arthroplasty, exacerbates the burden on the individual and health care system [1–4]. As the number of surgeries surged yearly, the total number of PJI patients increased [5]. However, the diagnosis and treatment of PJI remain challenging for clinicians.

Current diagnostic methods of PJI include serological testing, synovial fluid testing, and intraoperative histological pathology [2, 5]. However, although the diagnostic criteria are well defined, no gold standard has yet been established [4, 6]. Similarly, treatment of PJI is difficult for clinicians because there are no widely accepted criteria [7, 8]. The two-stage revision is currently the standard procedure for PJI, but the proper time to perform the second-stage revision is still debatable [9].

As recent literature revealed the close correlations between the coagulation cascade and infection course, coagulation-related biomarkers are gaining attention. Some biomarkers, such as D-dimer and fibrinogen (Fib), have been proven promising for PJI diagnosis [10–13] and determining the reimplantation timing [10, 14].

Thromboelastography (TEG) is a routine coagulation test that assesses the whole process of clotting over time in the body [15] and provides a full-scale evaluation of clot formation, elasticity, and duration. Additionally, various coagulation elements are measured as follows [16]. The clotting time (K value) reflects the rate of blood clot formation and is an indicator of fibrinogen function [17]. The α-angle (angle) represents the clot growth rate, while the maximum amplitude (MA) is the maximum clot amplitude [18]. The amplitude at 30 min (A30) measures clot strength at 30 min after MA [19], and thrombodynamic potential index (TPI) was derived from the MA and K values [20].

Moreover, TEG yields information about all phases of coagulation and provides further information on standard coagulation tests [16, 21]. Numerous studies have proved that TEG is useful in evaluating coagulation status, predicting bleeding in patients with severe sepsis, monitoring haemostasis during cardiac surgery and liver transplant procedures, etc. [15, 21, 22]. However, no study has reported its value in diagnosing PJI and guiding the timing of reimplantation for the second-stage revision.

Therefore, this study aims to investigate (1) the value of TEG in distinguishing PJI from aseptic loosening and (2) the ability of TEG parameters to guide the proper time for the second-stage revision. Furthermore, the measured TEG parameters were compared with the ESR, CRP, and D-dimer levels.

## Methods

We conducted this retrospective study including all revision total hip and total knee arthroplasties performed in our hospital from October 2017 to September 2020, under the ethical approval of the institutional review board of our hospital. Among the 145 patient records acquired, 61 patients were diagnosed with PJI according to the Musculoskeletal Infection Society criteria [23], and 84 patients were diagnosed with aseptic loosening. Patients were excluded if at least one of the following are present: (1) lack of needed data, (2) recent use of anticoagulants, (3) presence of inflammatory arthritis such as rheumatoid arthritis and ankylosing spondylitis, (4) blood diseases such as thrombocytopenic purpura, (5) formation of deep vein thrombosis of the lower limbs, (6) liver diseases, (7) malignancy, and (8) infection of other tissues or organs.

Finally, 62 patients who underwent revision arthroplasty were included in this study: 23 in group A (treated for PJI) and 39 in group B (treated for aseptic loosening). A total of 83 patients were excluded due to lack of needed data (*n* = 55), deep vein thrombosis in the lower limbs (*n* = 24), recent use of oral warfarin due to coronary stent implantation (*n* = 2), urinary tract infection (*n* = 1), and rheumatoid arthritis (*n* = 1).

The patients’ fasting venous blood samples were collected routinely on the second day of admission and sent to the clinical laboratory of our hospital for blood examination, including routine blood examination, conventional coagulation tests, and TEG. The test results were acquired approximately 30 min after blood collection. Moreover, at least 3 tissue culture specimens (including joint fluid) were obtained when the participants underwent revision arthroplasty, and these samples were cultured for 7–14 days. Group B underwent a one-stage revision. Meanwhile, the two-stage revision for group A consisted of the following procedures: 1) The first-stage revision involved removal of the former prosthesis, supervened with implantation of antibiotic-loaded cement spacers (4 g vancomycin in 160 g gentamicin-containing bone cement). 2) At least 3 months after the first stage of treatment, surgeons decided whether to implant a new prosthesis or continue antibiotic protocols based on clinical symptoms and laboratory parameters. Aseptic patients were prescribed oral rivaroxaban 10 mg daily for 35 days for thromboprophylaxis, and PJI patients followed the same order after each staged surgery. All included patients were regularly followed up at 1 month, 3 months, 6 months, 1 year, and then each year after discharge. Functional outcomes, complications, and the reasons for any reoperation were recorded. According to Delphi-based consensus, success of reimplantation was defined by (1) control of infection, as characterised by a healed wound without fistula, drainage, or pain; (2) no subsequent surgical intervention for infection after reimplantation surgery; and (3) no occurrence of PJI-related mortality [24, 25].

### Statistical analysis

Statistical analysis was performed using IBM SPSS Statistics for Windows (version 26; IBM Corporation, Armonk, NY, USA), and statistical significance was set at *p* < 0.05. Independent sample t-tests were used for data conforming to normal distributions. In contrast, the Mann-Whitney U tests were used for data not conforming to normal distributions, and categorical variables were summarised using chi-squared tests. Receiver operating characteristic (ROC) curves were drawn using SPSS. The following parameters were calculated in each test: area under the curves (AUCs), sensitivity, specificity, positive predictive value (PPV), negative predictive value (NPV), positive likelihood ratio (+LR), and negative likelihood ratio (−LR). Moreover, we further studied the diagnostic value of the different combinations of mentioned parameters in pairs. The Youden index was used to determine the optimal cut-off value of these biomarkers for the diagnosis of PJI. Furthermore, a scatterplot was drawn using GraphPad Prism (version 8.0.2; GraphPad Software)8.

## Results

The demographic characteristics of each group are shown in Table 1. There were no statistically significant differences between baselines of the two study groups, except for involved joints. Particularly, the hip joint accounted for 87.18% in group B and 30.43% in group A, which was statistically significant (*p*<0.01).

**Table 1:** Demographics of the Study Groups

Demographics	Group A (*n* = 23)	Group B (*n* = 39)	*p* Value
Age (y)^a^	64.13 ± 9.10	64.56 ± 10.88	.873
Sex^b^			. 424
Male	10 (43.48%)	13 (33.33%)	
Female	13 (56.52%)	26 (66.67%)	
BMI (kg/m^2^) ^a^	26.16 ± 2.83	25.55 ± 3.61	.493
Involved joint^b^			<0.01
Hip	7 (30.43%)	34 (87.18%)	
Knee	16 (69.57%)	5 (12.82%)	
Serum inflammatory and fibrinolytic markers			
CRP (mg/L)	45.71 ± 54.50	3.57 ± 4.62	<.05
ESR (mm/h)	57.59 ± 27.97	14.59 ± 10.33	<.001
D-dimer (mg/L)	1.72 ± 1.22	1.06 ± 1.23	<.001
K (min)	1.26 ± 0.77	1.56 ± 0.41	<.001
Angle(°)	72.56 ± 7.38	67.80 ± 6.13	<.001
MA (mm)	71.23 ± 5.62	62.83 ± 4.03	<.001
A30(mm)	70.85 ± 5.79	62.36 ± 4.16	<.001
TPI(/sec)	126.28 ± 54.46	60.30 ± 26.24	<.001

*BMI* body mass index, *SD* standard deviation, *CRP* C-reactive protein, *ESR* erythrocyte sedimentation rate, *K* clotting time, *Angle* α-angle, *MA* maximum amplitude, *A30* amplitude at 30 min, *TPI* thrombodynamic potential index

^a^ The values are expressed as the mean ± SD

^b^ The values are expressed as the numbers of patients, with the percentage in parentheses

We observed significant differences in tested markers between Groups A and B (*p* < .001), as shown in Table 1. The median values of CRP, ESR, D-dimer, and TEG parameters (Angle, MA, A30, TPI) in group A were significantly higher than those in group B (*p* < .001). In contrast, the median K value was lower in group A (*p* < .001).

**Fig. 1:**
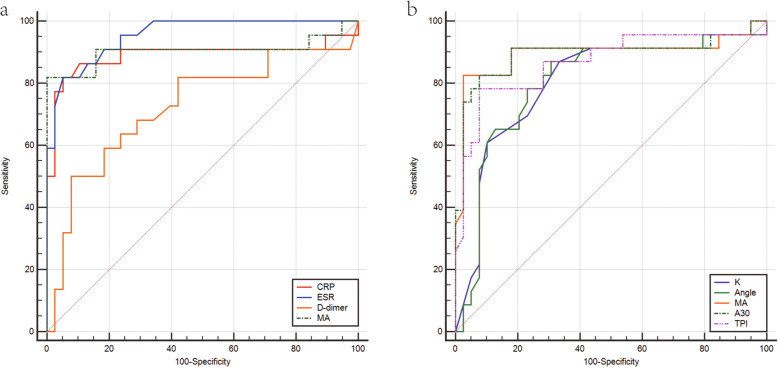
The comparison of ROC curves. ROC, receiver operating characteristic curve. CRP, C-reactive protein; ESR, erythrocyte sedimentation rate; MA, maximum amplitude; K, clotting time; Angle, α-angle; A30, amplitude at 30 min; TPI, thrombodynamic potential index

To evaluate and compare the diagnostic value of the tested markers, ROC curves of each inflammatory and fibrinolytic marker are illustrated in Fig. 1, while the AUC of each ROC curve was calculated (Table 2). The biomarkers’ optimal cut-off values for the diagnosis of PJI are shown in Table 2. The AUCs for CRP, ESR, and D-dimer were 0.893 (95% confidence interval [CI], 0.787–0.958), 0.953 (95% CI, 0.866–0.991), and 0.717 (95% CI, 0.588–0.824), respectively. The AUCs of TEG parameters ranged from 0.800 (K value, 95% CI, 0.680–0.891) to 0.895 (MA, 95% CI, 0.790–0.958). Among all tested biomarkers, ESR had the highest AUC, while D-dimer had the lowest AUC. The AUC of MA ranked first among the TEG parameters, followed by A30, TPI, Angle, and K value. Although the AUC was lower than that of ESR, MA achieved a better sensitivity and specificity.

**Table 2:** The Diagnostic Value of Inflammatory and Fibrinolytic Markers

Markers	AUC	Optimal cutoff	Youden index	SEN (%)	SPE (%)	PPV (%)	NPV (%)	+LR	-LR
CRP (mg/L)	0.893	>8.8	0.7735	82.61	94.74	90.5	90.0	15.70	0.18
ESR (mm/h)	0.953	>34.0	0.7669	81.82	94.87	90.0	90.2	15.95	0.19
D-dimer (mg/L)	0.717	>1.7	0.4013	47.83	92.31	78.6	75.0	6.22	0.57
K (min)	0.800	<1.3	0.5362	86.96	66.67	60.6	89.7	2.61	0.20
Angle(°)	0.803	>70.7	0.5619	86.96	69.23	62.5	90.0	2.83	0.19
MA (mm)	0.895	>68.1	0.8004	82.61	97.44	95.0	90.5	32.22	0.18
A30(mm)	0.893	>66.5	0.7492	82.61	92.31	86.4	90.0	10.74	0.19
TPI(/sec)	0.867	>84.4	0.7057	78.26	92.31	85.7	87.8	10.17	0.24

*AUC* area under the curve, *SEN* sensitivity, *SPE* specificity, *PPV* positive predictive value, *NPV* negative predictive value, *+LR* positive likelihood ratio, *−LR* negative likelihood ratio, *CRP* C-reactive protein, *ESR* erythrocyte sedimentation rate, *K* clotting time, *Angle* α-angle, *MA* maximum amplitude, *A30* amplitude at 30 min, *TPI* thrombodynamic potential index

The diagnostic values of the different combinations of tested parameters in pairs are shown in Table 3. The combination of TEG parameters and CRP/ESR led to improved AUC, sensitivity, and specificity, except for ESR + MA and ESR + A30. In addition, CRP + A30 achieved an obvious boost in the diagnosis value.

**Table 3:** The Diagnostic Value of Combined Inflammatory and Fibrinolytic Markers

Markers	AUC	SEN (%)	SPE (%)	PPV (%)	NPV (%)	+LR	-LR
CRP + ESR	0.980	100.00	89.47	84.6	100.0	9.50	0.000
CRP + K	0.943	91.30	94.74	91.3	94.7	17.35	0.092
CRP + Angle	0.946	91.30	94.74	91.3	94.7	17.35	0.092
CRP + MA	0.995	100.00	94.74	92.0	100.0	19.00	0.000
CRP + A30	0.999	100.00	97.37	95.8	100.0	38.00	0.000
CRP + TPI	0.968	91.30	97.37	95.5	94.9	34.70	0.089
ESR + K	0.958	86.36	94.87	90.5	92.5	16.84	0.140
ESR + Angle	0.953	86.36	94.87	90.5	92.5	16.84	0.140
ESR + MA	0.948	90.91	89.74	83.3	94.6	8.86	0.100
ESR + A30	0.948	81.82	97.44	94.7	90.5	31.91	0.190
ESR + TPI	0.955	81.82	97.44	94.7	90.5	31.91	0.190

*AUC* area under the curve, *SEN* sensitivity, *SPE* specificity, *PPV* positive predictive value, *NPV* negative predictive value, *+LR* positive likelihood ratio, *−LR* negative likelihood ratio, *CRP* C-reactive protein, *K* clotting time, *Angle* α-angle, *MA* maximum amplitude, *A30* amplitude at 30 min, *TPI* thrombodynamic potential index, *ESR* erythrocyte sedimentation rate

The culture results of 23 patients with PJI are listed in Table 4, wherein 19 (82.6%) patients had positive results. Gram-positive bacteria, particularly *S. aureus*, are the most common pathogens observed. According to Table 5, the values of CRP, ESR, MA, A30, and TPI in Fungi group were less than those of the other three groups (G+ group, G- group and negative group).

**Table 4:** Culture Results of PJI Patients

Classification	Strain	Number of patients
G+	*S. aureus*	3
	*S. epidermidis*	2
	*S. dysgalactiae*	1
	*S. agalactiae*	1
	*S. intermedius*	1
	*Viridans Streptococci*	1
	*F. magna*	1
	*C. glutamicum*	1
G-	*E. coli*	2
	*Pseudomona aeruginosa*	1
	*Brucella*	1
	*Alcaligenes*	1
Fungi	*C. parapsilosis*	2
	*Aspergillus flavus*	1
Negative		4
Total		23

*G+* Gram-positive bacteria, *G-* Gram-negative bacteria

**Table 5:** Serum Inflammatory and Fibrinolytic Markers in PJI Patients with Different Pathogens

Markers	G+ (*n* = 11)	G-(*n* = 5)	Fungi(*n* = 3)	Negative(*n* = 4)
CRP (mg/L)	59.82 ± 55.58	45.40 ± 83.25	15.00 ± 2.27	30.33 ± 15.73
ESR (mm/h)	64.78 ± 35.07	55.20 ± 25.33	49.00 ± 32.51	55.75 ± 16.26
D-dimer (mg/L)	1.71 ± 1.28	1.33 ± 0.89	1.33 ± 0.77	2.52 ± 1.64
K (min)	1.46 ± 1.08	0.94 ± 0.17	1.33 ± 0.06	1.03 ± 0.17
Angle(°)	70.74 ± 10.24	75.74 ± 1.68	70.93 ± 0.71	74.83 ± 2.70
MA (mm)	70.63 ± 7.47	72.08 ± 2.61	68.73 ± 2.79	73.70 ± 3.82
A30(mm)	70.43 ± 7.58	71.58 ± 3.27	68.37 ± 2.64	72.98 ± 4.69
TPI(/sec)	117.06 ± 60.90	141.20 ± 36.25	81.63 ± 12.94	166.45 ± 51.92

*SD* standard deviation, *CRP* C-reactive protein, *ESR* erythrocyte sedimentation rate, *K* clotting time, *Angle* α-angle, *MA* maximum amplitude, *A30* amplitude at 30 min, *TPI* thrombodynamic potential index

The values are expressed as the mean ± SD

**Fig. 2:**
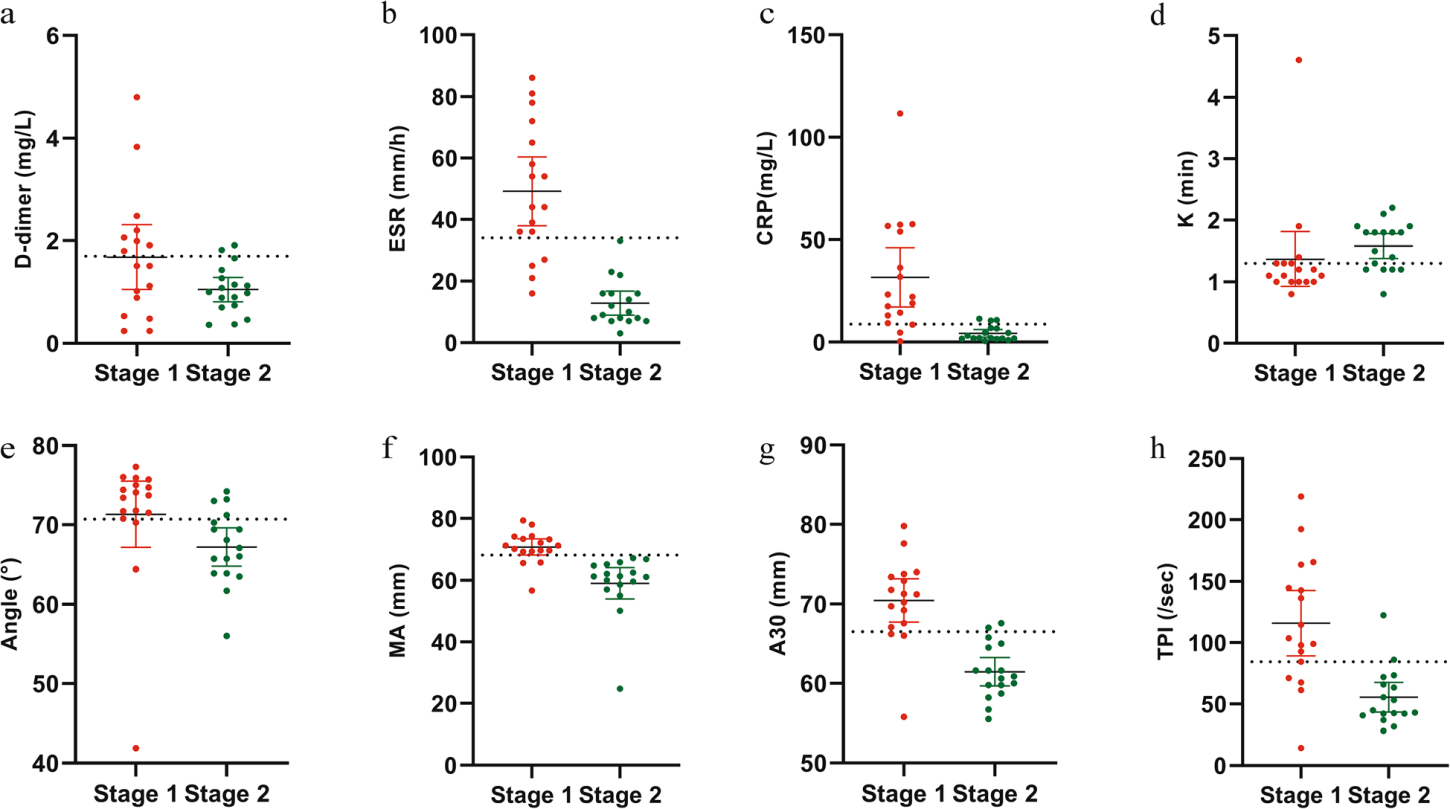
The distributions of D-dimer (**a**), ESR (**b**), CRP (**c**), K (**d**), Angle (**e**), MA (**f**), A30 (**g**), and TPI (**h**) levels before and after the first staged revision surgery. Stage 1: Stage before spacer insertion. Stage 2: Stage after re-admission for the second stage. Dotted lines represent the optimal cutoff values based on the present study. ESR, erythrocyte sedimentation rate; CRP, C-reactive protein; K, clotting time; Angle, α-angle; MA, maximum amplitude; A30, amplitude at 30 min; TPI, thrombodynamic potential index

Data from 17 patients who underwent two-stage revision surgery were available in the history database management system. The other six patients could not be re-admitted for the second-stage surgery due to short intervals from the first-stage surgery (less than 3 months). According to Delphi-based consensus, no patients showed a failure of reimplantation during the follow-up period (17.29 ± 8.29 (range, 3–28) months). However, 2 of 17 patients had poor knee function with limited range of motion. On comparing the tested biomarkers between stage 1 (the stage before spacer insertion) and stage 2 (the stage after re-admission for the second-stage surgery) of PJI patients, all tested markers except for D-dimer (*p* = 0.059) were found statistically significant. Furthermore, the numerical values of ESR and MA decreased below the cut-off value during stage 2 (Fig. 2) when each patient’s parameters were compared with corresponding cut-off values.

## Discussions

Many studies have revealed a close association between coagulation and infection. Endotoxins or components of the bacterial cell wall were reported to trigger changes in coagulation through tissue factor (TF) released by a variety of cells, such as vascular endothelial cells and monocytes, and maintain TF at high levels by stimulating the release of cytokines, such as interleukins and tumour necrosis factor α [21, 26, 27]. Correspondingly, coagulation-related biomarkers have recently been proven to be valuable in PJI diagnosis. D-dimer has been adopted as a minor criterion in the 2018 International Consensus Meeting criteria for PJI, but it has raised a huge controversy. Yan et al. [28] conducted a meta-analysis and observed that D-dimer is an effective serum biomarker for PJI diagnosis in patients without a history of hypercoagulation or inflammatory arthritis. In contrast, Tejbir et al. [2] and Rui Li et al. [29] reported the poor diagnostic value of D-dimer for PJI. Meanwhile, Lauren et al. [30] found that D-dimer results vary significantly in different laboratories, even for the same sample. Thus, D-dimer as a PJI diagnosis criterion was refuted. In our study, D-dimer exhibited a low value in PJI diagnosis, and its numerical values remained high after ESR and CRP levels were decreased in the normal range. Moreover, these conventional coagulation biomarkers can only reflect quantitative changes in platelet and fibrinogen levels.

TEG can provide comprehensive coagulation status of our body and provide additional data compared with standard coagulation tests [21]. Many studies have compared TEG and conventional coagulation tests in many clinical fields. For example, Hani et al. [16] demonstrated that TEG provides more information about the haemostatic state of patients with cirrhosis than conventional coagulation tests. Furthermore, Luo et al. [31] reported that TEG may be a reliable alternative to conventional coagulation methods for diagnosing sepsis-induced coagulopathy.

To date, no study has compared the TEG’s value with the three most used biomarkers (CRP, ESR, and D-dimer) in diagnosing PJI. In the present study, we highlight the value of ESR in diagnosing PJI, with AUC, optimal cut-off, sensitivity, and specificity for ESR of 0.953, 34.0 mm/h, 81.82, and 94.87%, respectively. D-dimer had the lowest AUC, and it remained high in PJI patients before the second-stage surgery. Although there were small differences in the numerical values of AUC, cut-off value, sensitivity, and specificity, our results are similar to those of several other studies [11, 12, 29]. Additionally, our study found that MA achieved a good diagnostic value with a specificity of 97.44%. The combination of CRP/ESR with TEG parameters (K, Angle, MA, A30, TPI) achieved higher sensitivity and specificity than any individual marker, except for the two combinations (ESR + MA, ESR + A30). Thus, despite lower AUCs of these parameters than ESR, TEG remains a promising diagnostic test for PJI.

Performing the second-stage revision in proper timing is the key to boosting the success rate of PJI treatment. Hence, researchers continue exploring the optimal timing for reimplantation. In recent years, various indicators have been developed. Hoell et al. [32] reported that CRP was not a reliable parameter to exclude persistent infections. Tao Bian et al. [33] also concluded that ESR and CRP were of limited value in determining the reimplantation timing by pooled analysis. Meanwhile, some studies report that coagulation-related biomarkers perform well in guiding reimplantation. Shahi et al. [14] highlighted D-dimer in determining the optimal timing of reimplantation. Moreover, Geng Bin et al. [10] reported fibrinogen as a useful tool for assessing infection outcomes after first-stage surgery. However, a small sample of both studies limited their credibility.

Furthermore, we found that ESR and MA were good indicators for determining the timing of reimplantation. Our study showed a 100% success rate of two-stage revision, which is higher than those reported in most published studies. Several reasons contribute to this result: 1) the follow-up time of 4 patients was shorter than 1 year, which may not be enough to judge infection control. 2) Although widely adopted by many researchers, the Delphi-based consensus is not the gold standard for evaluating the success of the two-stage revision, as it overlooks the functional outcomes of surgery. Therefore, our success rate would be lower when the functional outcome is considered.

According to the results of this study, TEG has the following advantages. First, as a regular and routine serological test for coagulation, the TEG assay does not incur additional costs or suffering to patients. Second, the TEG parameters may be applied to differentiate PJI from aseptic loosening, particularly the combination of CRP and MA/A30. Finally, MA/A30 appears to be a valuable tool for assessing infection control after spacer insertion.

Our study has several limitations. First, the samples in this study were small; a larger sample size might have produced different results. Second, lower extremity Doppler ultrasound was routinely performed to exclude venous thromboembolism of the lower limb, which did not rule out clots in other parts of the patient’s body. Moreover, we did not consider PJI patients’ use of antibiotics before admission to our hospital. Furthermore, this study has some inherent biases due to its retrospective nature. Finally, we only checked the TEG of PJI patients before spacer insertion and before reimplantation, rather than checking them regularly. Hence, the changing trend of the biomarker levels in PJI patients was not clear.

## Conclusion

This study reports 5 measured TEG parameters (K value, angle, MA, A30, TPI) that are statistically different between patients with PJI, with aseptic loosening, and those who were re-admitted for reimplantation in two-stage arthroplasty. With high specificity, MA was considered a valuable biomarker in diagnosing PJI and assessing infection control after the first-stage surgery.

## Data Availability

The datasets used and/or analyzed during the current study are available from the corresponding author on reasonable request.

## Abbreviations

PJI: Periprosthetic joint infection
TEG: Thromboelastography
MSIS: Musculoskeletal Infection Society
CRP: C-reactive protein
ESR: Erythrocyte sedimentation rate
K: Clotting time
Angle: α-angle
MA: Maximum amplitude
A30: Amplitude at 30 min
TPI: Thrombodynamic potential index
Fib: Fibrinogen
ROC: Receiver operating characteristic curves
AUCs: The areas under the curves
PPV: Positive predictive value
NPV: Negative predictive value
+LR: Positive likelihood ratio
-LR: Negative likelihood ratio
CI: Confidence interval
TF: Tissue factor
ILs: Interleukins
TNFα: Tumor necrosis factor α
ICM: International Consensus Meeting
